# Functional MRI of Native and Non-native Speech Sound Production in Sequential German-English Bilinguals

**DOI:** 10.3389/fnhum.2021.683277

**Published:** 2021-07-19

**Authors:** Miriam Treutler, Peter Sörös

**Affiliations:** ^1^European Medical School Oldenburg-Groningen, Carl von Ossietzky University of Oldenburg, Oldenburg, Germany; ^2^Department of Neurology, Carl von Ossietzky University of Oldenburg, Oldenburg, Germany; ^3^Research Center Neurosensory Science, Carl von Ossietzky University of Oldenburg, Oldenburg, Germany

**Keywords:** articulation, bilingualism, vowel, syllable, sensorimotor cortex, cerebellum, inferior frontal cortex, insula

## Abstract

Bilingualism and multilingualism are highly prevalent. Non-invasive brain imaging has been used to study the neural correlates of native and non-native speech and language production, mainly on the lexical and syntactic level. Here, we acquired continuous fast event-related FMRI during visually cued overt production of exclusively German and English vowels and syllables. We analyzed data from 13 university students, native speakers of German and sequential English bilinguals. The production of non-native English sounds was associated with increased activity of the left primary sensorimotor cortex, bilateral cerebellar hemispheres (lobule VI), left inferior frontal gyrus, and left anterior insula compared to native German sounds. The contrast German > English sounds was not statistically significant. Our results emphasize that the production of non-native speech requires additional neural resources already on a basic phonological level in sequential bilinguals.

## Introduction

Bilingualism and multilingualism, the ability to communicate in two or more languages, are highly prevalent. Although an exact definition of bilingualism and precise statistics are missing, it is estimated that more than 50% of the global population actively use more than one language (Bialystok et al., 2012). At least 55 countries have two or more official languages^1^. Many individuals are exposed to and use two languages on a daily basis from birth or starting in their first years of life (simultaneous or early bilinguals). Many others learn at least one foreign language (L2) at school or later in life (sequential or late bi- or multilinguals). In the European Union, 95% of all students in upper secondary education learn English as a foreign language, 22% Spanish, 18% French, and 17% German^2^.

With the advent of non-invasive methods of brain research, such as event-related potentials (ERPs), positron emission tomography (PET), and functional MRI (FMRI), the neural correlates of bilingual speech and language production became readily accessible to scientific research, contributing to the extensive increase of published studies on bilingualism in the past two decades (Kroll and Navarro-Torres, 2018). These studies showed convincingly that bilingualism is associated with a reorganization of neuronal networks related to speech-language production and cognitive control (Simmonds et al., 2011; Cargnelutti et al., 2019).

Numerous studies demonstrated that L2 production relies on neural systems that are also used in monolinguals, with often increased brain activity for L2 production due to cross-linguistic interference during lexical retrieval, articulatory planning, articulation, and auditory and sensory feedback (Del Maschio and Abutalebi, 2019). Most work on the organization of the bilingual brain has been performed on the word and sentence level (Sabourin, 2014; Kroll et al., 2015). In an FMRI study on sequential French–English bilinguals, overt sentence reading in L2 was associated with increased activity in the left inferior frontal gyrus, left premotor cortex, and left fusiform gyrus, compared to reading in L1 (Berken et al., 2015). In another FMRI study on sequential Japanese-English bilinguals, activity in the dorsal part of the left inferior frontal gyrus was correlated with L2 fluency in an English sentence production task (Shimada et al., 2015). Similarly, reading aloud isolated words or performing picture naming in L2 (and even in L1) was associated with increased activity in the precentral gyrus, pars triangularis, pars opercularis, anterior insula, superior temporal gyrus, and planum temporale, all of the left hemisphere, in bilinguals compared to monolinguals (Parker Jones et al., 2012). Considerably less research has been done on the production of non-lexical speech. Moser et al. (2009) investigated the production of three-syllable non-words that contained English or non-English syllables in native speakers of English and found increased activity for non-English syllables in several brain areas, including the left inferior frontal gyrus and the left anterior insula.

The exact mechanisms that cause the differences in brain activation between L1 and L2 production in bilinguals have not been determined so far (for a discussion, see Simmonds et al., 2011; Nichols and Joanisse, 2016). Two not mutually exclusive theories have attracted attention. One influential theory emphasizes the observation that neural plasticity in the speech-language network decreases during childhood and adolescence. This theory is heavily influenced by the notion of a critical period in speech-language acquisition, first proposed by Lenneberg (1967). As a consequence, L2 acquisition in sequential bilinguals occurs when the speech-language system is less plastic and less capable of establishing efficient neural networks. Less efficient neural networks are believed to require additional neural resources within the core speech-language system and in associated networks, such as executive control (Sulpizio et al., 2020). Another theory emphasizes the fact that most bilinguals are more proficient in L1 compared to L2. It is often assumed that less proficient participants activate more neural resources than proficient participants when performing a certain motor or cognitive task. This notion is, e.g., supported by a study on brain activity before and after learning to play a melody on a keyboard (Chen et al., 2012). Participants with the best performance after training showed less activity in the premotor cortex during playing compared to less proficient players. Of note, a growing body of literature suggests that both mechanisms, age of acquisition and proficiency, independently influence the brain activity during bilingual speech-language production (Oh et al., 2019).

We have previously investigated the production of speech sounds of different complexity frequently used in the participants’ native language with clustered FMRI acquisition (or sparse sampling) (Sörös et al., 2006, 2011). We found that the production of an isolated vowel (“a”), a consonant-vowel syllable (“pa,” “ta,” or “ka”), and a trisyllabic utterance (“pataka”) was associated with the activation of a distributed neural network of cortical and subcortical brain regions, including the primary sensorimotor cortex, the supplementary motor area, the cerebellum, and the superior temporal gyrus. The production of the more complex “pataka,” as compared to “a,” resulted in increased activity in the left inferior frontal gyrus, the left cerebellar hemisphere, and the bilateral temporal cortex (Sörös et al., 2006). This core network for speech motor planning, programming, and execution has been confirmed by various studies using functional neuroimaging and electrophysiology (for reviews, see Kemmerer, 2015; Tremblay et al., 2016).

In the present study, we investigated the production of isolated native and non-native speech sounds in sequential German-English bilinguals using FMRI. We studied the production of speech sounds commonly used in German (but unknown in English) and speech sounds commonly used in English (but unknown in German) in German university students who grew up in a monolingual German-speaking family and started to learn English at school. We used continuous fast event-related FMRI, after our pilot measurements demonstrated moderate head motion during overt speech production, corroborating the results of a recent FMRI study on overt sentence production (Berro et al., 2020). We hypothesized that production of non-native speech sounds should resemble the production of native, more complex sounds, i.e., should be associated with increased activity in key areas of speech motor control (such as the left inferior frontal gyrus and the cerebellar hemispheres).

## Methods

### Participants

For the present study, 15 healthy young adults were investigated. As two participants had to be excluded because of incorrect task performance (see section “Behavioral Data Analysis”), the following data analyses are based on 13 participants (seven women, six men) with a mean age ± standard deviation of 25.5 ± 3.0 years (minimum: 20 years, maximum: 32 years). All participants were native speakers of German (native language, L1) and started to learn English at school after the age of 6 years (first foreign language, L2). Participants self-rated their English proficiency between the levels B1/B2 and C1, according to the Common European Framework of Reference for Languages^3^. B1 is considered intermediate, B2 upper intermediate, and C1 advanced proficiency of a foreign language. According to the Edinburgh Handedness Inventory–Short Form (Veale, 2014), nine participants were right-handed (handedness scores: 62.5–100) and four participants were bimanual (handedness score: 50). All participants considered themselves as right-handed.

This experiment was part of a larger project on oral and speech language functions. Detailed inclusion and exclusion criteria for the participants were published in a paper on the neural correlates of tongue movements (Sörös et al., 2020). In brief, all participants were part of a convenience sample of students of the Carl von Ossietzky University of Oldenburg, Germany, without a history of neurological or psychiatric disorders or substance abuse. All participants gave written informed consent for participation in the study. A compensation of 10 € per hour was provided. The study was approved by the Medical Research Ethics Board, Carl von Ossietzky University of Oldenburg, Germany (2017-072).

### Experimental Paradigm

During the experiment, participants were visually cued to articulate one of the following four vowels or syllables (the symbols of the International Phonetic Alphabet^4^ are given in brackets): “ö” [ø:], “aw” [ɔ:], “che” [çə], and “the” [ðə]. The vowel “ö” and the syllable “che” are common in German, but do not exist in standard English. By contrast, the vowel “aw” and the syllable “the” are common in English, but do not exist in German. Especially the English “th” [ð] is notoriously difficult to pronounce for Germans and is usually spoken with a characteristic German accent.

The corresponding letters were projected onto a screen with an LCD projector and presented to the participants in the scanner through a mirror on the head coil using Cogent 2000 v125^5^ run in MATLAB R2015b.

**FIGURE 1:**
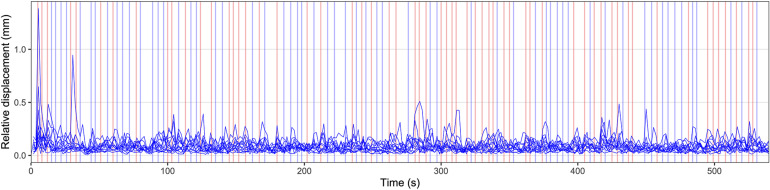
Illustration of the experimental paradigm and individual head motion over time. Relative displacement, the distance between one volume and the following volume, is displayed for all individual participants. Values for relative displacement start after the acquisition of the first brain volume, 1800 ms after the start of the measurement. The presented data represent the head motion before motion correction using volume realignment. The appearance of visual stimuli is marked by vertical lines. Blue vertical lines indicate a German stimulus (“ö” or “che”), red lines indicate an English stimulus (“aw” or “the”). Before the first visual stimulus and after the last visual stimulus rest periods of 5000 ms and 15000 ms duration, respectively, were included.

Using a fast event-related design, 120 visual stimuli (30 per condition) were shown in a pseudorandomized order for 1000 ms. The duration of the interstimulus interval was jittered between 2000 and 8000 ms. Between the presentation of visual stimuli, a fixation cross was presented in the middle of the screen. The experiment started with a rest period of 5000 ms and ended with another rest period of 15000 ms. During these two rest periods, the fixation cross was presented as well. The experimental paradigm is illustrated in Figure 1.

Before the FMRI experiment, a short training run was presented on the stimulation PC outside the scanner to give all participants the opportunity to familiarize themselves with the paradigm. All participants were instructed that German (“ö” and “che”) and English speech sounds (“aw” and “the”) need to be produced in a pseudorandomized order and that they should articulate the corresponding sounds as soon as the letters appeared on the screen in the loudness of a regular conversation. In addition, participants were told to keep their head as still as possible.

After the experiment described here, three additional experiments were performed during the same imaging session, including the overt production of tongue twisters, movements of the tongue (Sörös et al., 2020), and overt production of sentences. The duration of the entire scanning session was approximately 45 min.

### MR Data Acquisition

Structural and functional MR images of the entire brain were acquired on a research-only Siemens MAGNETOM Prisma whole-body scanner at 3 Tesla (Siemens, Erlangen, Germany) and a 64-channel head/neck receive-array coil located at the Neuroimaging Unit, School of Medicine and Health Sciences, Carl von Ossietzky University of Oldenburg, Germany^6^. We used a T1-weighted MPRAGE sequence to acquire structural data and a T2^∗^-weighted BOLD sequence (305 volumes, time of acquisition: 9:16 min) to acquire functional data (for details, see Sörös et al., 2020). Foam padding within the head coil was used to minimize head motion. All subjects wore noise-canceling wax ear plugs for hearing protection.

### Audio Recording and Noise Reduction

During the FMRI experiment, all utterances were recorded on a PC through an FMRI-compatible microphone attached at the head coil (FOM1-MR, Micro Optics Technologies Inc., Middleton, WI, United States).

The resulting sound files were processed after the measurement with the audio software Audacity^7^. Spectral noise gating was performed with Audacity’s *Noise Reduction* function to reduce the continuous gradient noise during the recording (Inouye et al., 2014). First, the gradient noise during the initial 5000 ms rest period (without verbal responses) was selected. The frequency spectrum contained in this sample was identified by Audacity using Fourier analysis. Second, the entire recording was selected and this frequency spectrum was effectively suppressed. The processed sound files contained clearly intelligible verbal responses, allowing us to control every single response and identify individual errors. However, analyses of exact speech onset latencies or detailed phonetic analyses were not possible.

### Behavioral Data Analysis

Relative (volume-to-volume) and absolute (relative to the middle volume) head motion were determined by volume-realignment using MCFLIRT (FSL version 6.00, Jenkinson et al., 2002).

All audio recordings were checked for correct task performance. One participant misunderstood the task and spoke all sounds twice, another participant pronounced all sounds considerably longer than demonstrated during the pre-scan training. Both participants were excluded from the further data analysis. The remaining 13 participants produced four wrong or unintelligible sounds (coughing); these individual sounds were also excluded from the FMRI data analysis.

### Preprocessing of Functional Images

Analyses of magnetic resonance imaging (MRI) data were done on Carl von Ossietzky University of Oldenburg’s high-performance computer cluster CARL. Preprocessing was performed using the preprocessing pipeline fMRIPrep version 20.0.5 (RRID:SCR_016216)^8^ (Esteban et al., 2019). In fMRIPrep functional data were motion corrected by volume-realignment using MCFLIRT and registered to the MNI152NLin6Asym standard space template. ICA-based Automatic Removal Of Motion Artifacts (AROMA)^9^ was used to denoise the functional images, using the non-aggressive option (Pruim et al., 2015). Slice time correction was not performed. Preprocessing reports for all participants are available at the Open Science Framework at https://osf.io/t9qcw/. After initial preprocessing, all data sets were spatially smoothed with a Gaussian kernel of 5 mm full width at half maximum (FWHM).

### Independent Component Analysis

To identify brain networks associated with speech sound production in our experiment, tensorial independent component analysis (ICA; Beckmann and Smith, 2005) was performed as implemented in MELODIC (Multivariate Exploratory Linear Decomposition into Independent Components, version 3.15; part of FSL version 6.00). Preprocessed data sets were pre-whitened (Woolrich et al., 2001) and projected into a 53-dimensional subspace using probabilistic Principal Component Analysis where the number of dimensions was estimated using the Laplace approximation to the Bayesian evidence of the model order (Beckmann and Smith, 2004). All data sets were decomposed into sets of vectors which describe signal variation across the temporal domain (time-courses), the session/subject domain and across the spatial domain (maps). Estimated independent component maps were divided by the standard deviation of the residual noise and thresholded by fitting a mixture model to the histogram of intensity values (Beckmann and Smith, 2004). Of note, for this model-free analysis the entire data sets were used; the results represent brain activity during both, L1 and L2 production.

### First-Level FMRI Analysis

Preprocessed functional data sets were analyzed with FEAT (FSL version 6.00), performing a general linear model-based time-series analysis using voxel-wise multiple linear regressions (Friston et al., 1995). All FMRI analyses were whole-brain analyses with adequate correction for multiple comparisons.

The time courses of the two German sounds and the two English sounds were convolved with a gamma hemodynamic response function (phase: 0 s, standard deviation: 3 s, mean lag: 6 s) and served as regressors of interest. The temporal derivative of each primary regressor was included as a regressor of no interest to improve the model fit to account for differences in response latency. Regressors of interest (experimental conditions) and regressors of no interest (temporal derivatives) formed the design matrix used for voxel-wise multiple linear regressions. Motion parameters and physiological noise regressors were not included in the design matrix because ICA-AROMA was used for denoising. To remove temporal autocorrelations, time-series pre-whitening was used (Woolrich et al., 2001).

After generating parameter estimates (PEs) for every primary regressor and every participant, the following contrasts of parameter estimates (COPEs) were calculated: (1) German > rest, (2) English > rest, (3) German > English, and (4) English > German. Z statistic images were thresholded non-parametrically using a cluster-forming threshold of *Z* > 2.3 and a (corrected) cluster significance threshold of *p* < 0.05. Brain activity maps of all 13 participants are available at https://osf.io/t9qcw/.

### Second-Level FMRI Analysis

Mixed-effects group analysis maps were generated by FLAME (stages 1 and 2) for all contrasts. Again, Z statistic images were thresholded at *Z* > 2.3 (*p* < 0.05). Brain activity maps for all contrasts are available at https://osf.io/t9qcw/.

Local maxima (peaks of brain activity) were identified within the Z statistic images using FSL’s cluster command (minimum distance between local maxima: 10 mm; 62 local maxima were found for the contrast English > German speech sound production). The anatomical location of each local maximum was determined with FSL’s atlasquery command and the following probabilistic atlases^10^ : (1) Harvard-Oxford cortical structural atlas (48 cortical areas), (2) Harvard-Oxford subcortical structural atlas (21 subcortical areas), and (3) Probabilistic cerebellar atlas (28 regions, Diedrichsen et al., 2009).

## Results

### Head Motion

Figure 1 displays the relative displacement between two adjacent MRI volumes for all participants. In one participant, three values >0.5 mm were found (1.38, 0.95, and 0.51 mm). In another participant, one value was 0.65 mm. All other values were less than 0.5 mm. The median relative displacement for all participants and all timepoints was 0.07 mm. The maximum absolute displacement between the middle volume as a reference and all other volumes was less than 3 mm in all participants (minimum: 0.26 mm, maximum: 2.89 mm).

### Brain Activity

**FIGURE 2:**
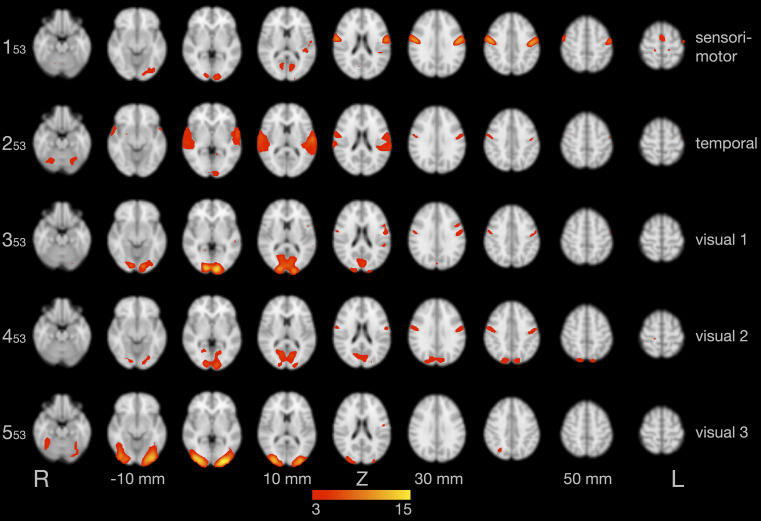
Results of the group independent component analysis (ICA). The five components with the highest explained variance are shown on axial slices in separate rows. Brain activity is color-coded in red and yellow. Images are in radiological convention (the left hemisphere is seen on the right).

Figure 2 illustrates the first five of 53 components of the tensorial ICA, a model-free group analysis combining all 13 data sets, which were already denoised by ICA-AROMA (as described in section “Independent Component Analysis”). These components represent a sensorimotor component (component 1: explaining 6.6% of the total variance), a component in the temporal lobe (component 2: 6.2%), and three visual components (component 3: 5.9%; component 4: 5.3%; component 5: 3.6%). Additional networks were also found, such as the default mode network and executive control network, but are not shown here.

**FIGURE 3:**
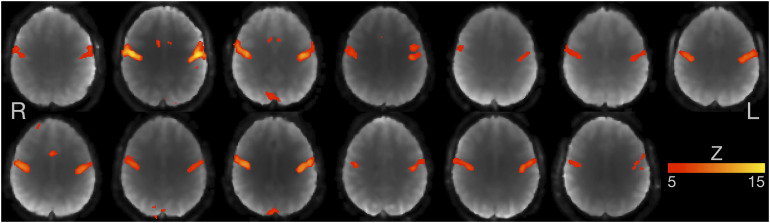
Individual FMRI results based on voxel-wise multiple linear regressions. Axial slices for all 13 participants are shown at the level of maximum activity in the primary sensorimotor cortex. Images are in radiological convention (the left hemisphere is seen on the right). *Z*-value ≥ 5.

An analysis using voxel-wise linear regressions based on the expected hemodynamic response (section “First-Level FMRI Analysis”) revealed that all participants showed similar and strong activity of the bilateral primary sensorimotor cortex (Figure 3; note: statistical threshold of *Z* ≥ 5).

The subsequent model-based group analysis (section “Second-Level FMRI Analysis”) showed that production of German and English speech sounds, compared to baseline, was associated with similar and widespread activation of cortical and subcortical areas, primarily related to speech motor control, phonological processing, and visual processing (individual and group data shown at https://osf.io/t9qcw/).

**FIGURE 4:**
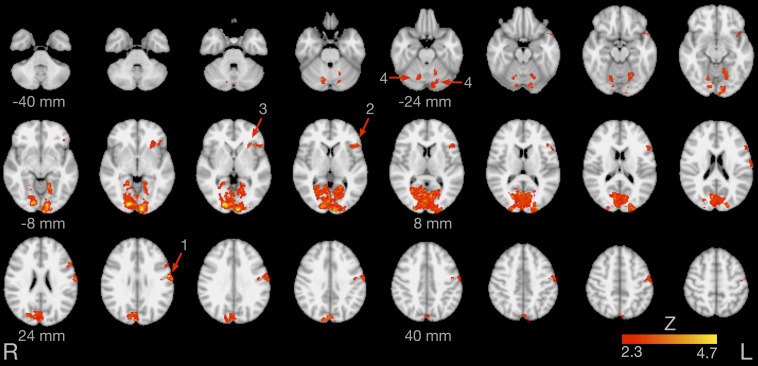
Brain activity for the contrast English > German speech sound production. The arrows point at the left lateral sensorimotor cortex (1), left inferior frontal gyrus (2), left anterior insula (3), and bilateral cerebellar lobule VI (4). Images are in radiological convention (the left hemisphere is seen on the right).

Figure 4 illustrates brain areas significantly more active during production of English compared to German sounds. These areas include key regions of speech motor control (left lateral sensorimotor cortex, left inferior frontal gyrus, left anterior insula, and bilateral cerebellar hemispheres). In addition, Figure 4 displays brain areas more active during visual processing of English compared to German cues, including the bilateral lingual gyrus and the bilateral occipital fusiform gyrus. Table 1 summarizes the coordinates of local maxima in MNI space and the respective *Z* value. The reverse contrast, German > English, did not result in significant differences.

**TABLE 1:** Local maxima of brain activity: stereotaxic coordinates in MNI space, *Z* values, and corresponding brain regions for the contrast English > German speech sound production.

Region	*x* (mm)	*y* (mm)	*z* (mm)	*Z* value
L precentral gyrus	−46	−12	32	3.24
L postcentral gyrus	−60	−6	28	4.37
L inferior frontal gyrus (triangular)	−48	26	4	3.70
L inferior frontal gyrus (opercular)	−54	14	26	3.53
L insula	−28	22	0	3.50
L cerebellum Lobule VI	−16	−58	−24	3.12
R cerebellum Lobule VI	14	−68	−28	3.59
L cuneus	−4	−86	22	4.75
R cuneus	6	−76	28	4.29
L intracalcarine cortex	−6	−80	6	3.98
R intracalcarine cortex	10	−76	16	4.65
L occipital pole	−8	−98	−2	4.61
R occipital pole	18	−88	−2	4.70
L lingual gyrus	−10	−88	−10	4.17
R lingual gyrus	4	−74	0	3.87
L occipital fusiform gyrus	−14	−82	−20	3.69
R occipital fusiform gyrus	24	−72	−6	3.76

## Discussion

The present fast event-related FMRI study on the overt production of German and English speech sounds in sequential German-English bilinguals demonstrated increased activity during non-native speech in critical areas of speech motor control. As speech production was cued by written letters, increased activity was also found in several occipital areas of the visual system (Figure 4 and Table 1). Speech production is a highly complex task, depending on several integrated processing stages. During speech production, the brain rapidly retrieves phonological information, executes speech motor programs, encoding movement trajectories of the articulators, and monitors continuously auditory and somatosensory feedback. These processing stages are materialized in a widespread articulatory brain network, including key areas of the pyramidal and non-pyramidal motor system, and in a phonological network, primarily located in the temporal lobes (Golfinopoulos et al., 2010; Hickok, 2014). An influential computational framework to describe the complex interplay between the phonological, motor, and somatosensory systems is the DIVA model (Guenther and Vladusich, 2012; Kearney and Guenther, 2019; Miller and Guenther, 2021).

### Areas of Increased Brain Activity

In the present study, production of non-native English sounds was associated with increased activity of the left primary sensorimotor cortex, bilateral cerebellar hemispheres (lobule VI), left inferior frontal gyrus, and left anterior insula.

The lateral primary sensorimotor cortex directly controls the muscles of the larynx (Brown et al., 2009; Simonyan and Fuertinger, 2015) and the articulators, including the tongue (Sörös et al., 2020), and processes somatosensory information of the oral cavity (Sörös et al., 2008). Although the laryngeal and orofacial midline muscles are innervated by both hemispheres, specific speech motor plans, or articulatory gestures, are primarily represented in the left primary motor cortex (Neef et al., 2015).

Functional MRI (Pulvermüller et al., 2006) and electrocorticographic recordings (Cheung et al., 2016) have demonstrated that the primary motor cortex is not only involved in speech production, but also in speech perception, presumably encoding distinctive phonetic features of individual speech sounds. Thus, increased activity of the left primary sensorimotor cortex during English vs. German speech sounds may also be related to the perception of the participants’ own voice (mainly through bone-conduction, as participants had noise-canceling ear plugs). Interestingly, we found increased activity during L2 production not only in the inferior part of the sensorimotor cortex (Figure 4, region 1), directly related to speech motor control, but also in more superior parts of the sensorimotor cortex. This more superior activity corresponds well to the results of electrocorticographic recordings reported by Cheung et al. (2016) during listening.

The cerebellar hemispheres receive afferents from the primary motor cortex via the cortico-ponto-cerebellar tracts and support sensorimotor control and coordination of laryngeal, orofacial, and respiratory movements (Ackermann, 2008). Generally considered to be heavily engaged in the rapid sequencing of speech sounds, forming syllables and words as well as producing the rhythm and intonation of continuous speech (i.e., prosody; Ackermann, 2008), the bilateral cerebellar hemispheres are also involved in the production of single vowels (Sörös et al., 2006). Multiple lines of evidence suggest that the cerebellar hemispheres are organized in a homuncular topology. Electric stimulation during neurosurgery demonstrated that the movements of the face and mouth are primarily represented in the hemispheric lobule VI (Mottolese et al., 2013). A recent high-resolution study on two individual subjects, investigating the functional connectivity between the cerebrum and the cerebellum using resting-state FMRI, corroborated this result (Xue et al., 2021). A graph theoretical analysis further elucidated the critical role of hemispheric lobule VI in the speech production network (Simonyan and Fuertinger, 2015).

The integrity of the left inferior frontal gyrus, although not part of the core motor system, has been linked to speech production since Broca’s seminal observations (Broca, 1861; Dronkers et al., 2007). The triangular and the opercular part of the left inferior frontal gyrus and, based on recent cytoarchitectonic and receptorarchitectonic analyses, adjacent frontal regions are the structural correlates of Broca’s area (Zilles and Amunts, 2018). In addition to its critical role in the left-hemispheric language network, Broca’s area is also believed to be part of the articulatory network (Fedorenko and Blank, 2020). Direct cortical surface recordings in neurosurgical patients suggested that Broca’s area mediates the information flow between the temporal cortex, the likely anatomical substrate of phonological planning, and the primary motor cortex, thus preparing an appropriate articulatory code to be executed by the motor cortex (Flinker et al., 2015). Deactivation of Broca’s area is associated with slowing of speech production (Long et al., 2016; Leonard et al., 2019).

The insulae are areas of sensory, motor, cognitive, and affective integration, e.g., processing somatosensory (Sörös et al., 2008; Pugnaghi et al., 2011) and nociceptive information (Fazeli and Büchel, 2018). The insulae are also involved in movements such as breathing (Herrero et al., 2018), swallowing (Sörös et al., 2009), and speech production (Ackermann and Riecker, 2010; Oh et al., 2014). The exact functions of the insulae in the articulatory network are under debate and still not entirely clear (Woolnough et al., 2019). Of note, an FMRI study on healthy individuals identified the left insula as an area associated with speech accent processing (Ghazi-Saidi et al., 2015).

The present study was designed to investigate the articulatory and phonological networks underlying L2 production. As we presented letters to cue verbal responses, we also found activity in parts of the visual system. We saw increased activity for the letter strings “the” and “aw” in the fusiform and lingual gyri, areas involved in letter recognition and orthographic to phonological mapping (Price, 2012; Murphy et al., 2019). Similarly, sentence reading in sequential bilinguals was associated with increased activity in the left fusiform gyrus when reading L2 compared to L1 (Berken et al., 2015).

Unexpectedly, we did not find differences between L1 and L2 production in the temporal cortex. Given the small sample size, we might have missed differences in phonological processing between conditions.

### Mechanisms of Increased Brain Activity

Several studies compared speech motor control during the production of L1 utterances of different complexities (Bohland and Guenther, 2006; Sörös et al., 2006; Riecker et al., 2008; Brendel et al., 2011) and found increased activity of the areas discussed above. In the present study, however, the formal complexity of the produced speech sounds was identical in German and English (a single vowel and a consonant-vowel syllable each). The interpretation of our results is not straightforward because our participants were sequential multilinguals and less proficient in English than in German.

Focusing on proficiency, we may argue that the production of L2 speech sounds requires more resources on different levels of the articulatory network, because L2 production is not as over-learned as L1 and performed in a less automatic fashion. This explanation would lead us to predict that intense training of the required sounds would result in decreased activity in the articulatory network. This interpretation appears to be supported by Ghazi-Saidi et al. (2015), who trained native speakers of Spanish to pronounce French cognates (phonologically and semantically similar words across languages) in a native accent for 4 weeks. In a picture naming paradigm, the authors found increased activity for the contrast L2 > L1 only in a small area of the left insula, but not in other areas of the articulatory network.

Focusing on age of acquisition, by contrast, we may argue that our participants started to learn English when German speech production was already consolidated and deeply encoded in the articulatory network, resulting in less efficient articulatory gestures for English speech production after the maturation of the articulatory network. This notion would lead us to predict that simultaneous bilinguals should not differ in brain activity when producing speech sounds in one of their languages. The notion of a sensitive period for speech motor control is corroborated by a study on sentence reading in simultaneous and sequential bilinguals, all using both languages on a daily basis (Berken et al., 2015). While brain activity was similar for simultaneous bilinguals, sequential bilinguals demonstrated increased activity in the left inferior frontal gyrus and left premotor cortex when reading aloud in L2 compared to L1. Importantly, activity in these areas showed a significant positive correlation with age of acquisition.

### Foreign Accent

The results of the present study may help to better understand the neural correlates of foreign accent. While simultaneous bilinguals usually speak in a native or native-like accent in their languages, most sequential bilinguals speak L2 with a foreign accent, even if they perform similar to natives on the lexical and grammatical level (Moyer, 2013). A foreign accent is characterized by deviations in pronunciation compared to the norms of native speech (Gut, 2009), mostly due to phonetic and phonological mismatches between L1 and L2 and caused by interference or transfer of pronunciation rules (Yavas, 2009). Our results imply that, for sequential bilinguals, the neural correlates of L2 production differ from L1 already at the fundamental level of vowel and syllable production and emphasize why it is so difficult, and often impossible, to loose a foreign accent.

### Methodological Considerations

Our study has four main limitations. First, the sample size (*n* = 13) is relatively small, compared to the recommendations for a typical task-based FMRI study (*n* = 30; Turner et al., 2018). Our sample size was not based on a formal sample size calculation because we were unable to find effect sizes for an FMRI experiment similar to ours. Rather, the sample size was based on resource constraints (Lakens, 2021), as the Neuroimaging Unit only allocated 15 h of measurement time to this non-externally funded project with four independent experiments. Nevertheless, we are convinced that our results are reproducible. Overt speech production is a particularly robust paradigm with relative little interindividual variability; Figure 3 illustrates the striking similarities in sensorimotor activity during speech sound production in our study across individuals. Moreover, we used a repeated-measures design comparing two conditions in the same participants rather than between two groups of participants and used a high-end Siemens MAGNETOM Prisma scanner. We also believe that it is important to report the results of studies with small sample sizes because our results may help researchers to perform sample size calculations for future, larger studies using, e.g., Neuropower^11^. Statistical maps for use in Neuropower can be found at https://osf.io/t9qcw/. Our peak coordinates (Table 1) may also be included in future voxel-wise quantitative meta-analyses with, e.g., GingerALE^12^ or Seed-based *d* Mapping^13^.

Second, our paradigm included visual stimulation (presentation of letters and letter strings), adding a reading component to this study initially designed to investigate overt speech sound production. In two previous studies we used auditory stimulation to cue overt speech production (Sörös et al., 2006, 2011). With this paradigm it was impossible to differentiate between the effects of auditory processing and phonological processing in the temporal lobe. This problem motivated the use of visual stimuli in the present study. Of course, we cannot rule out the possibility that visual letter recognition influenced brain activity in the areas of the speech network found here. Using independent component analysis, we were able to separate components with primarily sensorimotor, temporal lobe, and visual activity (Figure 2). However, the sensorimotor component also included visual activity and the temporal lobe and two of three visual components also included primary sensorimotor activity. Activity of the primary motor cortex was found in several studies on silent reading of action words (e.g., Pulvermüller et al., 2005). By contrast, the study by Tomasino et al. (2007) required silent reading of sentences describing an action with two different tasks, imagining the action and detecting a certain letter in the sentence. The contrast imagining > detecting involved speech motor areas, such as medial frontal cortex, thalamus, basal ganglia, and cerebellum, while the contrast detecting > imagining involved the visual and parietal cortex and the bilateral insula (Tomasino et al., 2007).

Third, our event-related design required frequent switching between L1 and L2. Again, we cannot definitively rule out the possibility that our results are influenced by language switching. In a voxel-wise meta-analysis, language switching was associated with activity in the left inferior frontal gyrus, left middle temporal gyrus, left middle frontal gyrus, right precentral gyrus, right superior temporal gyrus, midline pre-supplementary motor area, and bilateral caudate nuclei (Luk et al., 2011). Thus, it is unlikely that language switching accounts for all differences between German and English speech sound production seen in our study, but might have contributed to differences in activity of the left inferior frontal gyrus.

Finally, our study cannot explain potential mechanisms of the increased brain activity in English vs. German speech sound production found here. Due to the small sample size we were not able to investigate potential associations between brain activity and L2 proficiency.

### Recommendations for a Replication Study and Further Research

We recommend to perform a replication study, addressing the following methodological aspects.

#### Study Sample

Differences between L1 and L2 speech sound production should be tested in a larger sample, ideally after performing a sample size calculation based on the results of the present study.

#### Detailed Characterization of the Participants

We recommend to perform a standard language proficiency test, rather than relying on self-report, and to add a detailed questionnaire on language history and use, such as the Language and Social Background Questionnaire (Anderson et al., 2018). A detailed characterization of language history, use, and proficiency is important for the further investigation of the mechanisms that contribute to differences in L1 and L2 speech production.

#### Experimental Paradigm

We recommend to use a multiband T2^∗^-weighted imaging sequence, which was not available to us at the time of the present study. To control for potential effects of stimulation, we recommend to perform an event-related paradigm during (a) visual stimulation (reading letters) and (b) auditory stimulation (repeating pre-recorded speech sounds). To control for potential effects of language switching, we recommend to add a block-design experiment with less frequent language switching. A block-design would also minimize effects of stimulation, when the instruction is only given once, before the start of each block.

#### Data Analysis

Several studies have shown that the decision for a certain software package and analysis pipeline (Eklund et al., 2016; Olszowy et al., 2019; Botvinik-Nezer et al., 2020) and even the operating system used (Glatard et al., 2015) may affect the results of neuroimaging analyses. At least weak effects may not generalize across FMRI softwares and analysis strategies (Bowring et al., 2019). For a larger replication study we recommend to cross-validate effects with different, well-established analysis approaches. The reproducibility of these analysis approaches should be investigated as recommended previously (Sörös et al., 2021). One way to test the repeatability of the experiment and the analysis is to acquire two runs of the same paradigm and to compare the average of single runs before performing the grand average of all runs.

## Conclusion

The results of the present study on native and non-native speech sound production in sequential bilinguals add to our understanding of the neural correlates of bilingualism. While most studies on bilingual speech-language production focus on the word and sentence level, we are able to demonstrate that already the production of a non-native vowel and syllable is associated with increased activity in critical areas of speech motor control, such as the left primary sensorimotor cortex, bilateral cerebellar hemispheres (lobule VI), left inferior frontal gyrus, and left anterior insula.

## Data Availability Statement

The raw data supporting the conclusions of this article will be made available by the authors, without undue reservation.

## Ethics Statement

The studies involving human participants were reviewed and approved by the Medical Research Ethics Board, Carl von Ossietzky University of Oldenburg, Germany (2017-072). The participants provided their written informed consent to participate in this study.

## Author Contributions

MT was involved in conceptualization, data acquisition, data analysis, and contributed to the manuscript. PS was involved in conceptualization, data acquisition, data analysis, supervision, created the figures, and wrote the original draft of the manuscript. Both authors contributed to the article and approved the submitted version.

## Conflict of Interest

The authors declare that the research was conducted in the absence of any commercial or financial relationships that could be construed as a potential conflict of interest.

## Footnotes

1 https://www.uottawa.ca/clmc/55-bilingual-countries-world

2 https://ec.europa.eu/eurostat

3 https://www.efset.org/cefr/

4 https://www.internationalphoneticalphabet.org/ipa-sounds/ipa-chart-with-sounds/

5 http://www.vislab.ucl.ac.uk/cogent.php

6 https://uol.de/en/medicine/biomedicum/neuroimaging-unit

7 https://www.audacityteam.org

8 https://fmriprep.org

9 https://github.com/maartenmennes/ICA-AROMA

10 https://fsl.fmrib.ox.ac.uk/fsl/fslwiki/Atlases

11 http://neuropowertools.org/neuropower/neuropowerstart/

12 https://brainmap.org/ale/

13 https://www.sdmproject.com
